# Towards the Generation of an ASFV-pA104R DISC Mutant and a Complementary Cell Line—A Potential Methodology for the Production of a Vaccine Candidate

**DOI:** 10.3390/vaccines7030068

**Published:** 2019-07-18

**Authors:** Ferdinando B. Freitas, Margarida Simões, Gonçalo Frouco, Carlos Martins, Fernando Ferreira

**Affiliations:** 1 CIISA—Centro de Investigação Interdisciplinar em Sanidade Animal, Faculdade de Medicina Veterinária, Universidade de Lisboa, Avenida da Universidade Técnica, 1300-477 Lisboa, Portugal; 2 Laboratório de Virologia, Instituto Nacional de Investigação Agrária e Veterinária (INIAV), Quinta do Marquês, 2780-157 Oeiras, Portugal

**Keywords:** ASFV, pA104R, helper cell line, DISC, vaccine

## Abstract

African swine fever (ASF) is a fatal viral disease of domestic swine and wild boar, considered one of the main threats for global pig husbandry. Despite enormous efforts, to date, neither the classical vaccine formulations nor the use of protein subunits proved to be efficient to prevent this disease. Under this scenario, new strategies have been proposed including the development of disabled infectious single cycle (DISC) or replication-defective mutants as potential immunizing agents against the ASF virus (ASFV). In this study, we describe the methodology to generate an ASFV-DISC mutant by homologous recombination, lacking the *A104R* gene, which was replaced by the selection marker (*GUS* gene). The recombinant viruses were identified when the infected cells acquired a blue color in the presence of X-Gluc (100 µg/mL), which is the substrate for the *GUS* gene. Since these viral particles result from loss-of-function mutations, being unable to replicate, helper-cell lines expressing the viral pA104R protein were produced. Vero and COS-1 cell lines were transfected by different methods, both physical and chemical, in order to stably express the ASFV-pA104R. Best results were obtained by using Lipofectamine 2000 and Nucleofection methodology of Vero with the pIRESneo vector and by using Flp-FRT site-directed recombination technology system in Flp-In CV-1 cells (transformed COS-1 cells with a single integration site in a transcriptional active region). In order to ensure an efficient and stable integration of the viral ORF on the host cellular genome, the maintenance of the insert was verified by PCR and its expression by immunofluorescence and immunoblot analysis. Although the isolation of the recombinant virus was not achieved, the confirmation of ASFV-ΔA104R sequence, and the detection of the recombinant mutant through three passages, suggest that this approach is feasible and could be a potential strategy to generate safe and efficient DISC vaccine candidates.

## 1. Introduction

African swine fever (ASF) is an infectious disease of suids, with lethalities up to 100%. In addition, subacute forms, with reduced lethality (30% to 70%), as well as sub-clinical or chronic disease forms which can result in very low if any lethal cases, have also been described [1]. ASF is historically endemic in most of African Sub-Saharan countries and was first introduced into Europe via Portugal in 1957, afterwards spreading to neighboring countries, and to the Caribbean and Brazil. The disease was eradicated, except in Africa and on the island of Sardinia, where it remains endemic since 1978. From 2007, the disease was introduced in Georgia, Armenia, Russia and Iran, and later spread into Ukraine, Belarus, Baltic states, Poland, Czech Republic, Romania, Moldavia, Latvia and Hungary [1,2,3,4]. Recently, in 2018, ASF was reported in Belgium and China, and during 2019 in Vietnam [5,6]. So far, there is neither a vaccine nor a treatment against this global threat and the control of the disease relies on fast diagnosis, stamping out measures and trade bans of animals and pork products, giving rise to significant socio-economic losses in the affected areas [7,8].

ASF is caused by the African swine fever virus (ASFV), a large nucleocytoplasmic DNA virus being the sole member of the Asfarviridae family and *Asfivirus* genus [9,10,11]. Molecular genotyping identified at least 22 genotypes often linked to geographical distribution. The viral particle presents an icosahedral morphology, shaped by the capsid proteins [12,13], enclosing the inner viral core [14,15]. Extracellular virions have been recognized displaying an external layer, which is acquired from the host cellular membrane during the egress [15]. ASFV genome length is variable depending on the isolate (from 170–190 kbp), and encodes for more than 150 open reading frames (ORFs), from which, at least 50 have been characterized as structural proteins and others involved in regulatory processes, however many more lack known or predictable function.

The current ASF epidemic situation in Europe and Asia reveals the inability to contain the spread of the infection and consequently the eradication of ASF. Under this scenario, it is urgent to develop a safe and effective vaccine to prevent the dissemination of the disease [16]. Although efforts developed during the last 60 years, using different strategies to generate vaccines against ASFV as inactivated, subunit and live attenuated vaccines, have shown inability to induce levels of protection and safety needed to hamper this viral infection in pigs [16,17]; several factors are thought to contribute to the failure in generating vaccines such as the virus complexity, genetic variability and lack of knowledge on ASFV biology and virus–host interactions. The ASFV genome encodes among others, enzymes required for virion assembly, genome transcription and replication, including a putative histone-like protein, pA104R. This viral histone-like protein shares a sequence homology of 25% to 30% with two families of bacterial histone-like proteins (HU and IHF), being the only histone-like protein encoded by a eukaryotic virus [18,19]. These bacterial proteins are referred as nucleoid-associate proteins (NAPs), performing topological modifications of the chromosome (twisting, bending and folding), playing important structural and regulatory functions such as increasing the nucleoid stability [20]. Furthermore, these proteins show some similarities with eukaryotic histone proteins and are thought to act as architectural components within the nucleus, also modulating gene expression [21].

Recently, the role of ASFV-pA104R during infection was characterized in our lab [22]. Although the A104R transcripts were actively detected since 2 h post infection (hpi), the protein expression was reported only in the late phase of infection, colocalizing with the cell nucleus and viral factories. Moreover, the functional characterization of purified recombinant pA104R revealed that it binds to single-stranded DNA (ssDNA) and double-stranded DNA (dsDNA) over a wide range of temperatures, pH values and salt concentrations, and in an ATP-independent manner, with an estimated binding site size of about 14 to 16 nucleotides. Using site-directed mutagenesis, the arginine located in pA104R’s DNA-binding domain, at position 69, was found to be relevant for efficient DNA-binding activity. Together, pA104R and ASFV topoisomerase II (pP1192R) display DNA-supercoiling activity, although none of the proteins by itself does, indicating that both cooperate in this process. In addition, downregulation studies using small interfering RNA (siRNA) revealed that pA104R plays a critical role in viral DNA replication and gene expression, with transfected cells showing a diminished viral progeny (up to −82.0%), lower copy numbers of viral genomes (−78.3%), and a reduced number of transcripts of a late viral gene (−47.6%) [22]. Altogether, these results on pA104R suggest that it plays a critical role in viral DNA replication, transcription and packaging, emphasizing that ASFV mutants in the *A104R* gene could be used as a strategy to develop a vaccine candidate against ASFV.

As previously described, also for many other viruses (e.g., human immunodeficiency virus, herpes simplex virus, bluetongue virus), the deletion or the modification of crucial genes is being explored with the main goal of vaccine generation, mostly since the traditional strategies to produce a vaccine did not succeed [23,24,25]. These self-limited replication virions, also called disabled infectious single cycle particles (DISC), are expected to infect the host cells and express most of the early proteins similarly to the wild type virus. Nonetheless, replication will be aborted due to the lack of the essential protein expression. Most importantly, the inoculation of the full viral particle and subsequent protein expression is thought to be enough to trigger an immune response by stimulation of both major histocompatibility complex MHC classes I and II [26]. This strategy combines the safe advantage of the subunit vaccines with the strong immune stimulation of the host [25].

Altogether, the results herein presented were obtained in the effort to explore the possibility of generating a DISC virus based on the engineering of an ASFV mutant lacking the *A104R* gene, to be used as a vaccine candidate.

## 2. Material and Methods

### 2.1. Cell Line Cultures and Virus

Vero and COS-1 cells from the European Collection of Cell Cultures (ECACC) were maintained in Dulbecco’s Modified Eagle’s minimal essential medium (DMEM) supplemented with L-Glutamax, 10% fetal calf serum, inactivated (FCSI), 0.1% non-essential amino acids and antibiotic penicillin/streptomycin at 100 Unit/mL (all from Gibco, Life Technology, Karlsruhe, Germany). All experiments were conducted on actively replicating sub-confluent cells grown at 37 °C and under a 5% CO_2_ humidified atmosphere. When maintaining pIRESneo-ASFV ORF transfected cells, the medium was supplemented with the selection marker G418, to a final concentration of 200 µg/µL. 

Flp-In™-CV-1 cells work well with Flp-In™ vectors that express a gene under the control of a cytomegalovirus (CMV) promoter (pcDNA™5/FRT, pcDNA5/FRT/V5-His-TOPO^®^). This system (Thermo Fisher Scientific, Waltham, MA, USA) was designed for rapid generation of stable cell lines, expressing the protein of interest. These cells contained a single stably integrated FRT site, presumably at a highly active transcriptional region. Targeted integration of Flp-In™ expression vector ensured high-level expression of the viral gene of interest. Transfected cells were maintained with Zeocin™ Selection Antibiotic (500 µg/mL).

### 2.2. Viral DNA Extraction and A104R ORF Amplification

Extraction of viral DNA was performed with the High Pure Viral Nucleic Acid Kit (Roche, Mannheim, Germany), from the Vero-adapted Ba71V isolate. DNA fragments corresponding to *A104R* ORF were amplified by PCR using the following primers: A104R_Fw GGA CTA GTA TGT CGA CAA AAA AAA AGC CCA CAA TTA and A104R_Rev TCC CCC GGG TTA ATT TAA CAT ATC ATG AAC AGG TTT CAA TGC. A proof reading polymerase (Phusion^®^ High-Fidelity DNA Polymerase, Thermo Scientific) was used according to the manufacturer’s instructions (50 µL of master mix: 1 µL of forward and reverse primers (0.5 µM each), 10 µL of Phusion HF buffer, 1 µL dNTPs (200 µM), 0.5 µL Phusion DNA polymerase, 24.5 µL of Milli-Q water and 2 µL of DNA). All PCR reactions were performed in the iCycler Thermal Cycler PCR system (Bio-Rad, Munich, Germany), with the following thermal profile: initial denaturation at 98 °C for 1 min followed by 35 cycles of 10 s at 98 °C, 60–72 °C for 30 s and 72 °C for 30 s per kb), and final extension at 72 °C for 5 min. Following the PCR amplification, the obtained DNA fragments were cloned into different vector depending of the subsequent protocol. For practical reasons, each set of primers used to amplify the DNA fragments corresponding to the homology arms of A104R ORF and bacterial *GUS* gene (selection marker) are described in the following sections.

### 2.3. Plasmid Used for the Generation of Helper Cell Lines

The replication and isolation of the defective virus on the expected essential *A104R* gene was achieved by growth in a helper cell line that expressed ASFV-pA104R. The viral A104R ORF was inserted into the vector pIRESneo (Clontech, Mountain View, CA, USA), kindly provided by Günther Keil (Friedrich-Loeffler-Institut, Greifswald, Germany). The obtained fragment comprising the A104R ORF was phosphorylated with T4 polynucleotide Kinase (T4PNK from New England Biolabs^®^, Ipswich, MA, USA). The phosphorylation reaction (50 µL) included: 1 µL of DNA, 5 µL of 10× T4PNK reaction buffer, 5 µL of 10 mM ATP, 1 µL of T4 PNK (10 U) and water was incubated at 37 °C during 30 min, followed by heat inactivation at 65 °C for 20 min.

Plasmid pIRESneo linearization was carried out using: 1 µg of pIRESneo plasmid, 1 µL (10 U) of EcoRV restriction enzyme (New England Biolabs^®^), 5 µL of 10× NEBuffer and water were added (reaction volume of 50 µL). The mix was incubated at 37 °C (optimal temperature for EcoRV) for 60 min. Following this step, the linearized plasmid pIRESneo was dephosphorylated using Antarctic Phosphatase (New England Biolabs^®^). To 1 µg of linearized plasmid DNA, 10× Antarctic Phosphatase Reaction Buffer (1/10 of volume) and 1 µL of Antarctic Phosphatase (5 U) were added. The incubation step occurred at 37 °C for 15 min. Heat inactivation was performed at 65 °C for 5 min. 

Finally, the pIRESneo vector and insert (ORF A104R) ligation was performed using T4 ligase (Thermo Scientific) according to the manufacturer’s protocol. Since the linearization protocols adopted previously prompted the plasmid and insert with blunt ends, the reaction mixture (20 µL) for this procedure was prepared with 100 ng of linearized pIRESneo, 500 ng of the insert (5:1 ratio), 2 µL of Thermo Scientific 10× T4 DNA Ligase Buffer, 2 µL of 50% PEG 4000 solution, 5 U of T4 DNA Ligase and nuclease-free water. The incubation step was performed at 22 °C for 60 min. The generated plasmid was used to transform *Escherichia coli* DH5α upon antibiotic pressure. All the generated plasmids were isolated from bacteria using the High Pure Plasmid Isolation Kit (Roche Applied Science), following the manufacturer’s manual. To determine whether the cloned DNA fragments were incorporated into isolated vectors, their sequences were confirmed (including orientation by using an external primer recognizing the CMV promoter—Fw CGCAAATGGGCGGTAGGCGTG). Following this step, the concentration of each plasmid preparation was determined by spectrophotometric absorbance (NanoDrop, model 2000c) and finally stored at −20 °C until further use.

### 2.4. E. coli DH5α Transformation

Competent cells were thawed on ice and gently mixed with a pipette tip. About 50 µL of competent cells in a 1.5 mL Eppendorf was kept cooled on ice and added carefully to 5 µL of the phosphorylated construct. The transformation reaction was incubated on ice during 30 min, later subjected to a thermic shock of 42 °C for 30 s and placed on ice for 2 min. LB medium without antibiotic (250 µL) was added to each Eppendorf and placed on a shaker at 37 °C for an hour. Plates with LB medium and selection marker (ampicillin 50 µg/mL) were then inoculated with 100 µL of each mix and incubated at 37 °C overnight. Confirmation of the transformant cells was carried out through colony PCR using the primers referred previously.

### 2.5. Extraction, Linearization and Purification of the Construct

For the extraction of the construct from the *E. coli* DH5α cells, the EndoFree Plasmid Maxi Kit (Qiagen) was used. *E. coli* DH5α cultures were grown overnight on LB medium at 37 °C and harvested through centrifugation at 6000× *g* according to the Qiagen protocol. The NruI-HF (High Fidelity) Restriction Enzyme (New England Biolabs) was used to linearize the insert, following the manufacturer’s protocol. The digestion mix (50 µL) was prepared with: 1 µL (10 U) of NruI-HF, 1 µg of pIRESneo + ORF A104R, 5 µL of 10× NEBuffer and water, incubated at 37 °C (optimal temperature for NruI), for 60 min. To purify the construct, a non-toxic method based on ethanol precipitation was used, after restriction enzyme digestion. The volume of DNA was adjusted to 200 µL by adding sterile ddH_2_O plus 20 µL of 3 M sodium acetate (1/10 of the volume). Then, 2 volumes of 100% ethanol at –20 °C were added to the solution, vortexed for 10 s and placed on a –70 °C freezer for 20 min, subsequently centrifuged for 5 min and the ethanol supernatant was discarded, allowing the pellet to dry. DNA was resuspended in 20 µL of laboratory-grade water.

### 2.6. Transfection of Eukaryotic Cells

Vero and COS-1 cell lines were transfected using chemical and physical methods. For Lipofectamine 2000 (ThermoFisher) transfection the manufacturer’s protocol was followed. Briefly, cells were seeded on 6-well plates (2.5 × 10^5^ cells), growing O/N to be around 70–80% confluent when transfection was performed. Plasmid DNA–lipid complexes were prepared as: Lipofectamine 2000 reagent diluted in Opti-MEM Medium and 2.5 µg of the DNA construct diluted in 500 µL Opti-MEM (ThermoFisher), separately. Diluted DNA was added to Lipofectamine 2000 mixture on a 1:1 ratio and incubated for 5 min at room temperature. Next, the DNA–lipid complex was added to the cells and incubated at 37 °C for 1–3 days ongoing. For Lipofectamine LTX (ThermoFisher) transfection, 10 µL of Lipofectamine LTX was added to the diluted DNA and incubated at room temperature for 25 min for optimized DNA–Lipofectamine complex formation. Fresh DMEM added to cells and 500 µL of the DNA–Lipofectamine complex mix was added to each well, incubated at 37 °C under 5% CO_2_ during 24 h and medium refreshed. For JetPRIME^®^ (Polyplus-transfection, New York, NY, USA) transfection, cell cultures were prepared as for Lipofectamine 2000 transfection and the DNA–Oligo mixture was performed as recommended (2 µg of DNA construct was diluted in 200 µL of JetPRIME^®^ Buffer and mixed with the vortex). Then, 4 µL of JetPRIME^®^ was added to the solution, thoroughly mixed and after a short spin down, incubated at room temperature. Transfection mixture was added dropwise to each well (200 µL/well) and maintained at cell growing conditions. Four hours post-transfection, DMEM without antibiotics was replaced and cells incubated for 24 h before adding the selection marker Geneticin^®^ (0.5 mg/mL). Transfected cells were checked upon every day. As a procedure control and to confirm integration of the exogenous DNA insert, the plasmid pmaxGFP (Lonza, Basel, Switzerland) was transfected in Vero and COS-1 cells. For the physical Nucleofection method, the Amaxa Cell Line Nucleofector Kit V (Lonza) was used and the manufacturer’s protocol for COS-1 cells was followed. Briefly, COS-1 cells (8 × 10^5^ cells/cuvette) were resuspended in Nucleofector buffer and 2 µg of pIRESneo + ORF A104R was added. The cuvette was placed on the Nucleofector I device and the appropriate program was selected (Nucleofector^®^ Program D-05). Immediately after the Nucleofection process ended, cells were transferred into 6-well plates and incubated at 37 °C under 5% CO_2_. The selection marker was only applied after 24 h of post-electroporation incubation to avoid an excessive stress factor on cells. Vero cells were subjected to the same process.

**Figure 1:**
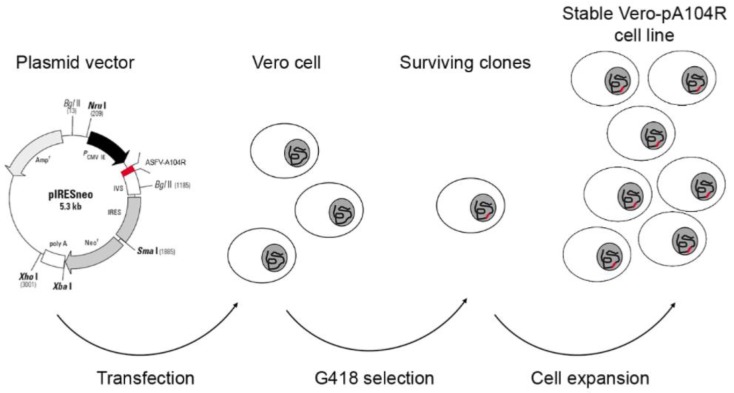
Schematic overview to generate helper cell lines expressing pA104R. Vero/COS-1 cells were transfected with the linearized pIRESneo_ASFV-A104R vector and then were subjected to antibiotic selective pressure. The surviving clones were expanded and the Vero-pA104R/COS-1-pA104R cell lines were established.

After the transfection, the surviving clones were expanded and stable Vero-pA104R and COS-1-pA104R cell lines were expanded after 40 repeated passages by using antibiotic selective pressure (1 mg/mL Geneticin). The strategy to obtain both cell lines is schematically described in Figure 1.

### 2.7. Genomic DNA Extraction from Transfected Cells

Genomic DNA was extracted by using the Quick-gDNA™ MiniPrep Kit (Zymo Research, Irvine, CA, USA) and following the manufacturer’s protocol. Transfected cell cultures were harvested after trypsinization and cell density was calculated by using a Neubauer chamber. Up to 5 × 10^6^ cells were selected and centrifuged at 600× *g* for 8 min. Supernatant was discarded and 500 µL of Genomic Lysis Buffer was added to the cell pellet. The solution was vortexed and let to stand for 10 min. The mixture was transferred to a Zymo-Spin Column in a collection tube and centrifuged. The supernatant was discarded and the column was placed in a new collection tube and DNA Pre-Wash Buffer was added to the spin column. The procedure was repeated as previously, but gDNA Wash Buffer was added instead. Another centrifugation was made, the supernatant was discarded and Elution Buffer was added to the spin column at RT. After incubation the column was centrifuged and the eluted DNA was ready to use or to store at −20 °C until further use.

### 2.8. Immunofluorescence Studies

Vero cell wild type, Vero-pA104R, COS-1 and COS-1-pA104R cells growing on glass coverslips, in 24-well plates (1.0 × 10^5^ cells), were fixed with 3.7% (*w*/*v*) paraformaldehyde in HPEM buffer (25 mM HEPES, 60 mM PIPES, 10 mM EGTA, 1 mM MgCl2) for 10 min and permeabilized with PBS/Triton X-100 (0.2%, *v*/*v*) for 5 min at RT. Cells were then washed with PBS, blocked with PBST and BSA (1%, *w*/*v*) for 30 min and incubated with an in-house anti-A104R polyclonal antibody for 1 h. After the wash step with PBS, the secondary antibody anti-mouse Alexa Fluor 588 (Abcam, Cambridge, UK) was also incubated for 1 h. All procedures were performed at room temperature (RT) and all antibody incubations were performed in a dark humidified chamber to prevent fluorochrome bleaching. Prolong© Gold Antifade/Vectashield mounting medium with DAPI (4′,6-diamidino-2-phenylindole, Vector Laboratories, Peterborough, UK) was used. Immunofluorescence analysis was performed using an epifluorescence microscope (Leica DMR HC model, Wetzlar, Germany), data sets were acquired by Adobe Photoshop CS5 software (Adobe Systems, Inc., San Jose, CA, USA) and images were subsequently processed with ImageJ open source software (version 1.46r).

### 2.9. Protein Extraction and Western Blotting

Vero cells wild type and Vero-pA104R cells were grown in 6-well plates (5.6 × 10^4^ cells/cm^2^). Before sampling, cells were washed twice with PBS and lysed in ice-cold modified RIPA buffer (25 mM Tris, pH 8.2, 150 mM NaCl, 0.5% *v*/*v* NP-40, 0.5% *w*/*v* sodium deoxycholate, 0.1% *w*/*v* SDS) supplemented with protease inhibitor cocktail (Complete, Mini, EDTA free; Roche) and phosphatase inhibitor cocktail (PhosStop; Roche). Clarified whole-cell lysates were further subjected to SDS-PAGE, using 8% to 16% (*w*/*v*) polyacrylamide separating gels, and transferred to a 0.2 μm-pore-diameter nitrocellulose membrane (Whatman; Schleicher & Schuell, Darmstadt, Germany) by electroblotting. For Tx solubility analysis, after the wash step with PBS, a buffer containing 50 mM HEPES (pH 7.6), 100 mM NaCl, 2 mM EDTA, 250 mM sucrose, 0.1% Tx supplemented with protease (Complete, Mini, EDTA free; Roche) and phosphatase (PhosStop, Roche) inhibitors were added to the cells. The blot membranes were blocked with PBST containing 5% (*w*/*v*) BSA (Sigma-Aldrich, St. Louis, MI, USA) for 1 h, at RT, and further incubated with specific primary antibodies (1 h at RT, in-house anti-pA104R, 1:100; anti-β-actin, 1:200, SC-69879; Santa Cruz Biotechnology, Dallas, TX, USA). Then, a secondary antibody conjugated with HRP was also incubated for 1 h at RT (anti-mouse IgG, 1:32,500, 1010-05; Southern Biotech, Birmingham, AL, USA). All the antibody incubations were followed by three 10-min wash steps with PBST, and protein detection was performed using a chemiluminescence detection kit (Pierce ECL Western blotting substrate; Thermo Scientific) on Amersham Hyperfilm ECL (GE Healthcare, Pittsburgh, PA, USA). β-actin was used as loading control.

### 2.10. Cell Cryopreservation

Transformed helper cell lines (stably expressing the viral gene) were preserved in a suspension of 80% DMEM, 10% FCSI and 10% dimethyl sulfoxide (DMSO), and cryovials were maintained in liquid nitrogen. The Vero-adapted ASFV-Ba71V isolate (Badajoz 1971) was used for all the infections and propagated as previously described [27]. Virus titres were determined by 50% tissue culture infectious dose (TCID50) titration using Vero E6 cells and the Spearman-Kärber method [28], and the infections were carried out at the indicated multiplicities of infection (MOI). After a 1 h adsorption period, the inoculum was removed and the cells were washed twice with serum-free medium.

### 2.11. Plasmids Used for Viral Gene Deletion

**Figure 2:**
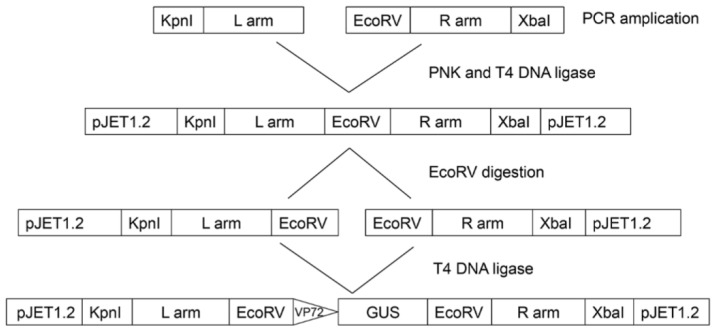
Schematic representation of the methodology used to generate the deletion plasmid vector. For the deletion of the *A104R* gene from the genome of Ba71V isolate, a plasmid vector pΔA104R_GUS containing the B646L (p72) promoter and GUS gene flanked by the homology arms of the *A104R* gene (L arm and R arm) was constructed.

A plasmid vector pΔA104R_GUS was constructed to support the deletion of the *A104R* gene from the genome of ASFV-Ba71V isolate. For this, the homology arms flanking the *A104R* gene were amplified from Ba71V genomic DNA. The left arm (L arm) corresponding to 500 bp (29,666–30,166) was amplified using the L arm_Fw (GGT ACC ATT TTT TAT GCC ATG G) and L arm_Rev primers (TCT TGT ATA AAA AAC TAA ATA TTT TTT TAT AAA AAT ATT AAA TCT ATT AAA A). Similarly, the right arm (R arm) corresponding to 504 bp (30,478–30,982) was amplified by PCR using the primers R arm_Fw (GAT ATC TTA AAT AAT AAA GCC ATC ATC ATC G) and R arm_Rev (TCT AGA AGT GAA TTT AGC GAG AAA T). To facilitate the insertion of the central region of the construct (B646L promotor and β-glucuronidase (*Gus*) gene) a restriction site was also introduced in the 5´end of the right arm. Following the amplification, the amplified L arm and R arm fragments were phosphorylated by T4 polynucleotide kinase (PNK, New England Biolabs) during 30 min at 37 °C and then ligated with each other by T4 ligase (Thermo-Scientific). The final total fragment was cloned into the vector pJet1.2 (Thermo-Scientific). This vector was then digested with EcoRV (New England Biolabs) overnight at 37 °C and dephosphorylated using Antarctic Phosphatase (New England Biolabs) during 60 min at 37 °C. The *GUS* gene from *Escherichia coli*, previously amplified using the PCR primers (GUS_Fw: ATT TAA TAA AAA CAA TAA ATT ATT TTT AT A ACA TTA TAT ATG TTA CGT CCT GTA GAA ACC CCA ACC; GUS_Rev: TCA TTG TTT GCC TCC CTG CTG) and phosphorylated, was inserted into the cut plasmid resulting in the vector pΔA104R_GUS (Figure 2). The pΔA104R_GUS vector included the GUS gene under the control of the B646L (p72) promoter flanked by adjacent regions of the *A104R* gene (L arm and R arm).

### 2.12. ΔA104R Recombinant Virus Production

**Figure 3:**
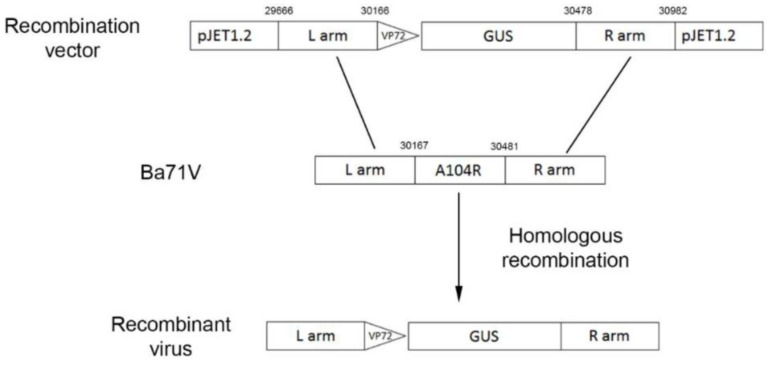
Schematic representation of the adopted strategy to generate the recombinant African swine fever (ASF) viruses. The target *A104R* gene was replaced by the reporter gene (*GUS*). The recombinant virus was obtained by homologous recombination between the left and right sequence regions (L arm and R arm) of the *A104R* gene on wild-type ASFV-Ba71V genome and recombination vector, resulting in the deletion of *A104R* gene and insertion of *GUS* gene under control of the strong viral promoter of the *ASFV-VP72* gene.

The protocol to generate the recombinant ΔA104R virus is based in the excision of the target gene and its replacement by the *GUS* gene through homologous recombination, as previously described for sequential deletion of *ASFV* genes [29] (Figure 3). Briefly, sub-confluent Vero cells (7.5 × 10^4^) were infected with ASFV-Ba71V isolate (MOI of 10) and incubated at 37 °C for 5 h in 24-well plates. Then the supernatant was removed and cells were washed twice with PBS (Gibco-Life Technologies). Prior to transfection protocol, the transfection mixtures containing 200 µL Opti-MEM (Gibco-Life Technologies), 2.5 µg pΔA104R_GUS vector and 5 µL TRANST-IT 2020 transfection reagent (Mirus) were homogenized, incubated at RT for 30 min and then added to the infected cells. After 4 h of incubation, cells were supplemented with 400 µL of DMEM and the incubation continued at 37 °C. The supernatant was collected at 48 h post-infection (hpi). In parallel, another protocol was also performed in which Vero cells are first transfected and then infected [30].

After these steps, the supernatants of the infected and transfected cells were used to infect new Vero cells on 6-well plates. Five hours after infection the virus inoculum was removed, the cells were washed with PBS and a 1% Agarose/DMEM overlay containing 5% FBS (all from Gibco-Life Technologies) and 100 µg/mL X-Gluc (5-bromo-4-chloro-3-indolyl-b-d-glucuronic acid, Sigma-Aldrich) was used to cover the cells. The recombinant viruses were identified by the blue appearance of the plaques. Individual blue plaques were picked, homogenized in DMEM, used to infect a new cell layer and subjected to additional plaque purifications.

Due to the technical difficulties, and in order to obtain a purified recombinant virus, infection at limiting dilutions was performed in Vero cells (96-well plates, 5 × 10^3^ cells/well). After 48 h, 100 µg/mL X-Gluc was added to the cells and, similar to the plaques method, wells containing recombinant GUS expressing viruses acquired blue appearance.

## 3. Results

### 3.1. Vero and COS-1 Cells Stably Express ASFV-pA104R

**Figure 4:**
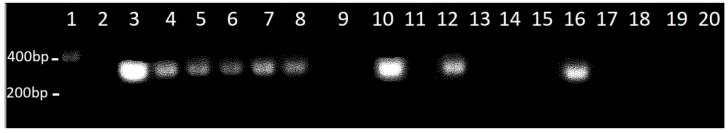
Open reading frame (ORF) A104R was successfully integrated in transfected Vero and COS-1 cells. ORF A104R was amplified by PCR (287 bp) from the genome of Vero-pA104R clones, cultured under antibiotic selective pressure (G418). Line 1: Molecular marker (NZYDNA Ladder III); Line 2: Vero wild type (wt, negative control); Line 3: pIRESneo A104R (positive control); Lines 4–6: three distinct Vero-pA104R clones, transfected with linearized plasmid and Lipofectamine 2000; Lines 7,8: two distinct Vero-pA104R clones, transfected with circular plasmid and Lipofectamine 2000; Line 9: COS-1 wt cells (negative control); Line 10: COS-1 cells transfected by Lipofectamine 2000 with the A104R construct; Line 11: Flp-In wt cells; Line 12: Flp-In cells transformed with the A104R construct; Lines 13–15: COS-1 cells transfected with Lipofectamine LTX (16 weeks); Line 16: FRT/A104R plasmid (positive control); Lines 17–20: Flp-In clones sustaining the selective pressure.

The establishment of a helper-cell line, expressing the viral protein encoded by the *A104R* gene, which was intended to be deleted, was mandatory to obtain and isolate a viral deletion mutant on this essential gene. Before transfection, Vero, COS-1 and CV-Flp-IN cells were plated under optimum conditions and following protocols tailored to each cell type and system/vector used. Six hours post-transfection culture media was changed, containing no cationic agents or selection antibiotics. At 48 h post-transfection, each transfected cell population was sub cultured and expanded, added with specific selective antibiotic, G418 for pIRESneo-based construct transfection and HygromycinB for cells transfected with the Flp-In/FRT system. Only cells stably transfected after a minimum of 16 consecutive weeks of selective pressure were considered for continued selection, screening, expansion and cryopreservation (Table 1). Cryopreserved cells, from each transformed cell line, were later thawed, subjected to selective pressure and after five passages, genomic extracts tested by PCR in order to confirm that the viral *A104R* gene was still integrated/present. Molecular assays by conventional PCR to confirm the integration of the viral gene in the transformed cell genome were performed regularly (Figure 4), and for this purpose, genomic DNA was extracted from resisting clonal populations (to selective pressure). Several transfection methodologies were used and results were diverse, with better survival and positive transfection in Vero pIRESneo system by using Lipofectamine 2000, regardless the linearization, but dependent on the plasmid vector.

The use of the Lipofectamine LTX was not as effective in Vero and COS-1 cells, as the JetPRIME^®^ transfection agent, but the latter method was significantly more cytotoxic inducing cellular death onset more rapidly. Finally, the Nucleofection of Vero cells was ineffective, with only COS-1 (linearized plasmid) able to grow and maintain the selective antibiotic pressure. Also regarding the two selected vectors (pIRESneo and pcDNA5-FRT) different outcomes were obtained as the latter was only available for COS-1 Flp-In cells. Still, concerning this Flp-In/FRT system, although prepared to achieve COS-1 Flp-In cells able to express the intended viral gene, with a V5/His tail, which would allow easier immunogenic detection, it had to be relegated, since no signal was obtained. Furthermore, the infection trials with distinct ASFV isolates revealed that these COS-1 Flp-In cells were only infected by the Ba71V isolate.

**Figure 5:**
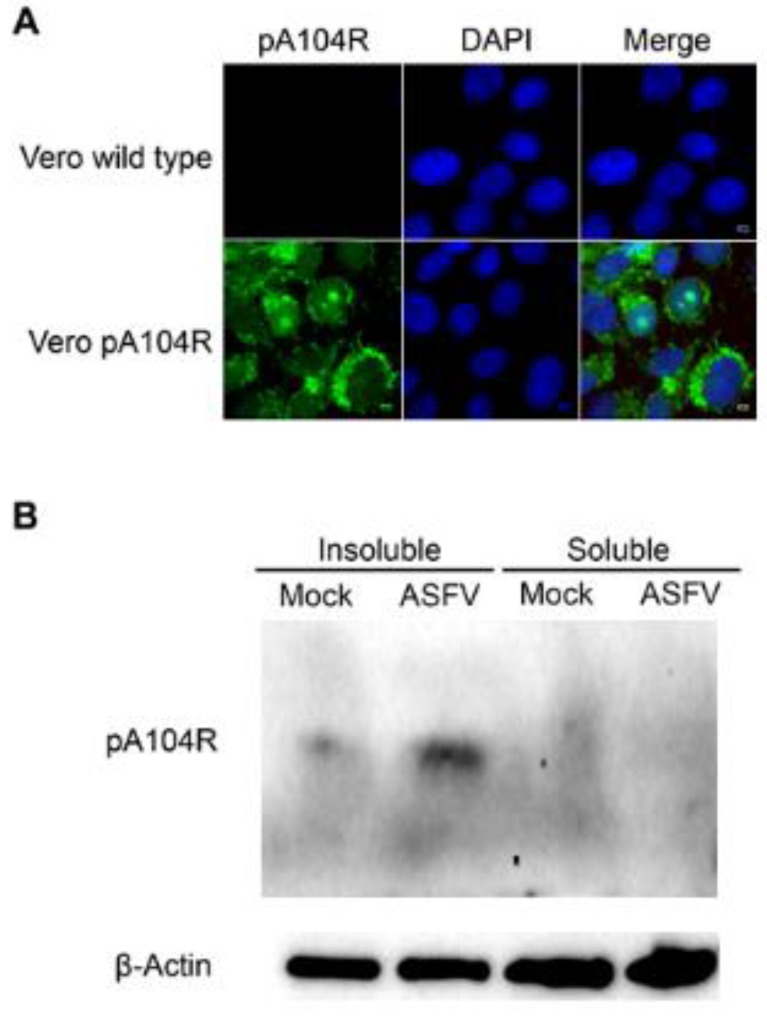
Helper Vero cells expressing viral pA104R. (**A**) Immunofluorescence studies detected African swine fever virus (ASFV)-pA104R in the cytoplasm and nucleus of Vero-pA104R cells. pA104R and DAPI (4′,6-diamidino-2-phenylindole) staining is shown in green and blue, respectively. (**B**) Transformed Vero cells non-infected and infected with Ba71V isolate (multiplicities of infection (MOI) = 2; 18 h post infection (hpi)) were re-suspended into buffer containing 0.1% Tx-100 and fractionated. pA104R was only detected in the Triton X-100-insoluble fraction, as expected, both in non-infected and infected transfected cells. β-actin was used as loading control.

**Figure A1:**
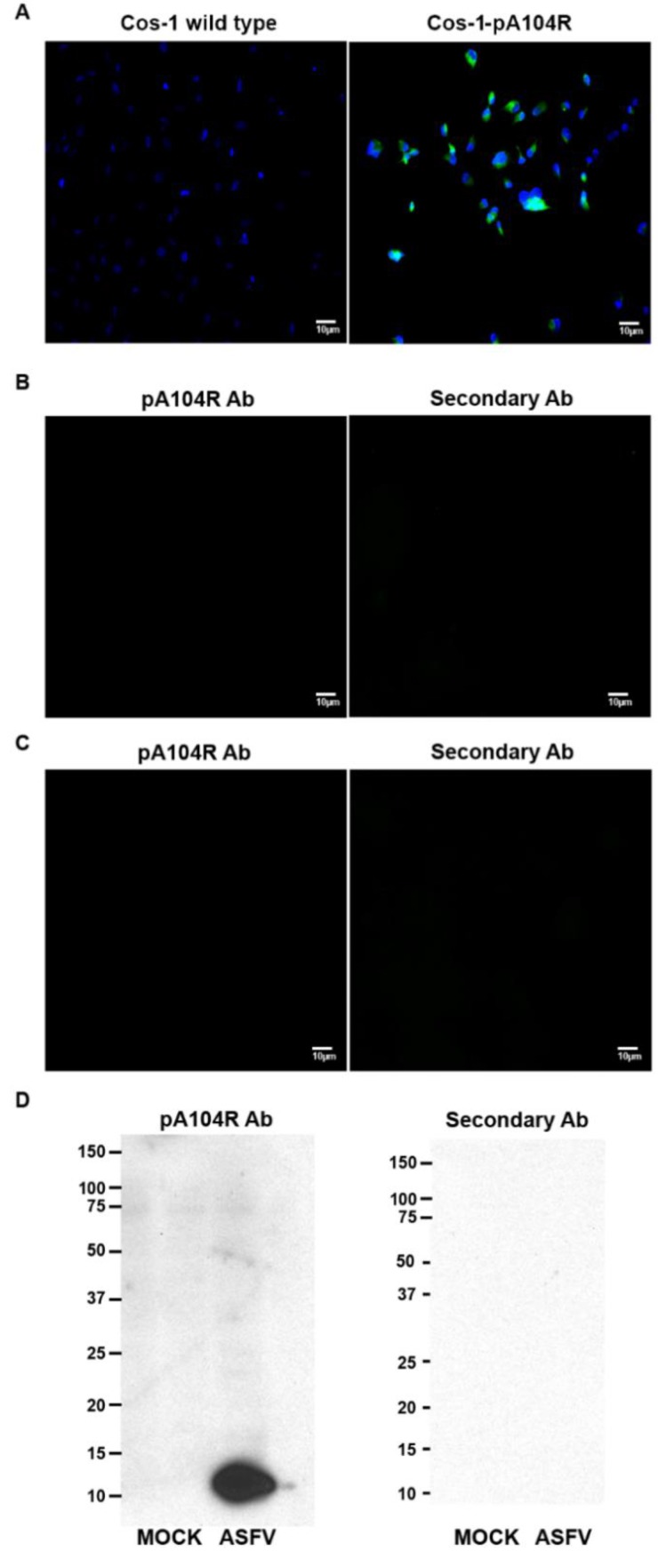
(**A**) Immunofluorescence studies showed ASFV-pA104R in the cytoplasm and nucleus of COS-1-pA104R cells (right image, green staining), in contrast with COS-1 wild type cells (left image). (**B**) Transformed Vero cells incubated with the in-house anti-pA104R antibody (left image) and with a goat anti-mouse secondary antibody (right image). (**C**) Transformed COS-1 cells incubated with the in-house anti-pA104R antibody (left image) and with a goat anti-mouse secondary antibody (right image). (**D**) Whole cell extracts of Vero cells non-infected and infected with Ba71V isolate (MOI = 2; 18 hpi) probed against the primary in-house antibody (anti-pA104R) and the secondary antibody (anti-mouse horseradish peroxidase-HRP).

To confirm the expression of the recombinant pA104R in the transformed cell lines, immunofluorescence and immunoblotting studies were also performed. Due to economic and technical reasons, further experiments were only performed in Vero cells. The viral histone-like protein was diffusely distributed throughout the cytoplasm and in some nucleus of Vero-pA104R cells (Figure 5A) and COS-1-pA104R cells (Appendix A Figure A1A). Controls are presented in Appendix A Figure A1B,C, respectively. As expected, the pA104R was only detected in Tx-insoluble protein fraction, showing a band with lower signal intensity when transformed Vero cells were compared with transformed Vero ASFV-infected cells (Figure 5B). Controls are depicted in Appendix A Figure A1D.

### 3.2. Deletion of the A104R Gene from the ASFV-Ba71V Isolate

Recombinant ASFV was generated by homologous recombination between the recombination vector pΔA104R_GUS (comprising the left and right homology arms flanking the *GUS* gene) and the genome of ASFV-Ba71V isolate, within infected Vero cells expressing the viral pA104R. Cells were infected with ASFV-Ba71V isolate prior to the transfection with the pΔA104R_GUS vector, and the supernatants were collected after 48 hpi. When supernatants were used to infected new cell layers in the presence of X-Gluc (100 µg/mL), some cells presented a blue color, meaning that the *ASFV-A104R* gene was replaced for the *GUS* gene by homologous recombination. Viral progeny was quantified by titration using helper Vero-pA104R cells, with the titers of recombinant virus being always much lower than the ones when compared with the titers obtained from the wild type virus. Aiming to obtain higher titer of the recombinant virus, the transfection of infected cells was repeated using different concentrations of plasmid and transfection agent and different multiplicities of viral infection. Additionally, another protocol was also tested, with Vero cells being first transfected and then infected, as previously reported [30]. However, regardless of the different procedures used, the ratio between viral yields from wild type ASFV and recombinant virus remained high, complicating the recombinant virus isolation.

**Figure 6:**
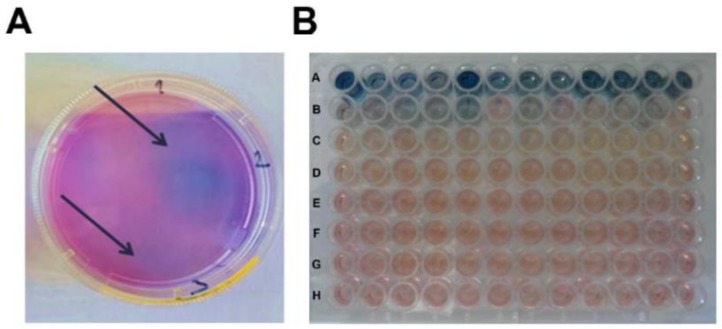
Isolation of recombinant virus using the plaques method. (**A**) Supernatants of the recombination step were used to infect new Vero cells expressing pA104R, which were covered by agarose with X-Gluc. The blue plaques formed were picked and used to infect new cell cultures. (**B**) Isolation of recombinant virus using serial dilutions. (A–E) Supernatants of the recombination step were diluted (1:10–1:100,000) in Dulbecco’s Modified Eagle’s minimal essential medium (DMEM) with X-Gluc and used to infect new Vero cells expressing pA104R. (F–H) The blue-colored supernatants were collected, diluted and used to infect new cells.

To isolate recombinant ASFV-ΔA104R mutant, supernatants obtained from recombination steps containing wild type and recombinant virus were used to infect new Vero-pA104R cell cultures, which were afterwards covered with agarose containing X-Gluc. After 72 hpi, some blue lytic plaques were found (Figure 6A), suggesting a productive cell infection by the recombinant virus mutants. Those plaques were collected, homogenized and passaged in new cell cultures. Considering that in the third passage no blue plaques were detected, a different isolation approach was conducted in cell cultures in 96 well plates, using various dilutions of the supernatants from the recombination assays (Figure 6B). Using this method, blue-colored cells (expressing β-glucuronidase) were observed up to the third passage of the initial supernatant stock.

## 4. Discussion

Traditionally, live-attenuated ASF viruses have been studied as vaccine candidates against ASF. Moreover, pigs infected with naturally occurring low virulent ASFV isolates, attenuated virus obtained by passages in cell cultures or viruses deleted in genes involved in virulence, develop resistance to the homologous virus, but rarely to heterologous isolates [31,32,33,34,35,36].

Nowadays, the unsuccessful efforts to develop an effective vaccine against ASF have been associated with lack of knowledge on molecular aspects of ASFV infection and immunity mechanisms [37]. In order to better characterize ASFV biology and to identify viral antigens that could induce a protective immune response in the pig, different ASFV proteins putatively involved in viral replication and transcription, namely pA104R, have been studied in our lab. pA104R, also known as histone-like protein, has an extensive sequence similarity to bacterial proteins that are implicated in genome replication and packaging [18,19]. Previous studies performed in our lab showed that pA104R, together with the ASFV-topoisomerase II (pP1192R), displays DNA supercoiling activity, suggesting that pA104R participates in the modulation of viral DNA topology and may be involved in viral DNA replication, transcription and packaging [22]. These results emphasize that the *A104R* gene may constitute a good target for deletion and to be used on a potential strategy to develop a DISC vaccine against ASF. Furthermore, siRNA-A104R assays demonstrated that this late viral protein is essential to the progression of ASFV infection, thus an A104R-defective ASFV mutant is expected to enter host cells, not being able to condense its genome, resulting in aberrant/immature ASFV particles, non-infectious, and incapable of producing a second cycle of infection.

Consequently, the production of a vaccine based on the above-mentioned model is expected to be safe and efficient in that viral antigens produced during the early and middle phases of infection cycle may induce a protective immune response in vivo. Taking these aspects into consideration, we proposed to generate an ASFV mutant lacking the ORF A104R. First, we generated several helper Vero/COS-1 cell lines stably expressing pA104R. However, lower expression levels of pA104R were detected in comparison to the wild-type Vero cells infected with ASFV-Ba71V isolate, suggesting that the amount of the pA104R supplied by Vero-pA104R could not be sufficient to support the replication of the ASFV-pΔA104R mutant. Indeed, the establishment of a functional helper-cell line is crucial to isolate and propagate a viral mutant lacking an essential gene for virus replication, assembly and morphogenesis. ASFV recombinant mutants were successfully obtained by homologous recombination, as demonstrated by the blue appearance of the supernatant from infected and pΔA104R_GUS-transfected cells, in the presence of X-Gluc. However, after three passages of blue-colored plaques or supernatants, in the isolation process of ASFV-ΔA104R using either the plaque method or limiting dilution, no recombinant mutants were detected, contrasting with the presence of wild type virus disclosed by observation of the cytopathic effect in non-blue-colored cells.

## 5. Conclusions 

Overall, our results suggest that the Vero cell line expressing pA104R may not fully support the replication of ASFV-pΔA104R recombinant virus, explaining why no blue-plaques could be found after a third passage. Indeed, the low expression of pA104R in these cells, and/or other unknown factors (e.g., incorrect folding, post-translational modifications) may not allow the replication of the recombinant virus. Indeed, the presence of blue-stained cells, at early steps of the isolation process, may be justified by one of two hypotheses: the recombinant virus is able to establish a first cycle of infection and the expression of *GUS* is enough to allow detection of blue-colored cells; or, since wild-type ASFV is present at higher concentrations in the early steps of isolation, it is possible that these viral particles co-infect the same cells, providing supplementary viral pA104R, thus enabling the replication of the recombinant virus, a mechanism described in other viruses [38].

Although the ASFV-ΔA104R mutant could not be isolated, propagated and further characterized, the work here described opens new insights for an innovative strategy to obtain ASFV mutants in essential proteins, a tool reinforced by the fact that a replication-defective or a single-cycle mutant virus may constitute an invigorating approach to control ASF, taking into consideration the safety issues regarding the vaccine development.

## Figures and Tables

**Table 1:** Cells and transfection methodology, progression and outcome.

CELLS	METHOD	AGENT	pIRESneo	48 h *	4 Weeks	16 Weeks
Vero	Chemical	Lipofectamine 2000	linearized	†	8 colonies	8 colonies
			circular	†	4 colonies	4 colonies
		Lipofectamine LTX	linearized	††	1 colony	-----------
		JetPRIME		†††	1 colony	-----------
	Physical	Nucleofection		††††	0 colony	-----------
COS-1	Chemical	Lipofectamine 2000	linearized	†	2 colonies	2 colonies
			circular	†	1 colony	1 colony
		Lipofectamine LTX	linearized	†	1 colony	0 colony
		JetPRIME		††††	0 colony	-----------
	Physical	Nucleofection		††	3 colonies	3 colonies

(*) Cellular death observed († = 25% death; †† = 50% death; ††† = 75% death; †††† = 100% death). Success of transfection evaluated by the number of colonies surviving selective pressure throughout the indicated weeks.
